# Assessing Self-Interaction Corrections in the Selective Catalytic Reduction of NO on a Cu-SSZ-13 Zeolite Cluster Model

**DOI:** 10.1021/acs.jpca.6c02902

**Published:** 2026-07-20

**Authors:** Priyanka B. Shukla, Selim Romero, Tunna Baruah, Rajendra R. Zope, Koblar A. Jackson, J. Karl Johnson

**Affiliations:** † Department of Chemical & Petroleum Engineering, 6614 University of Pittsburgh, Pittsburgh, Pennsylvania 15261, United States; ‡ Department of Physics, 12337 University of Texas at El Paso, El Paso, Texas 79968, United States; § Computational Science Program, 12337 University of Texas at El Paso, El Paso, Texas 79968, United States; ∥ Physics Department and Science of Advanced Materials Program, 5649 Central Michigan University, Mount Pleasant, Michigan 48859, United States

## Abstract

Self-interaction error (SIE) in density functional theory (DFT) calculations can lead to inaccurate descriptions of catalytic processes. In this work, we investigate the effects of SIE on a catalytic NO activation cycle on a cluster model of the Cu-SSZ-13 zeolite, systematically identifying transition states and reaction pathways for both single- and multistep reactions. We assess SIE using the Perdew–Zunger self-interaction correction (PZSIC) method, implemented with the Fermi-Löwdin orbital SIC (FLOSIC) approach. We benchmark the performance of standard DFT functionals (LDA, PBE, and r^2^SCAN) and SI-corrected functionals (PZSIC-LDA and the locally scaled LSIC­(*z*_σ_)-LDA) against CCSD­(T) reference energies for both adsorption energies and reaction barriers. The standard functionals increase in accuracy in the order LDA < PBE < r^2^SCAN, but all three tend to overestimate adsorption energies and underestimate reaction barriers. While PZSIC-LDA improves the description of reaction energetics over LDA for several reaction steps, it introduces large errors for processes that involve a change in the Cu-oxidation state. These errors are linked to the spurious destabilization of a filled 3d^10^ electronic shell relative to 3d^9^ in PZSIC calculations as described recently by Maniar et al. (Proc. Nat. Acad. Sci. 122, e2418305122 (2025)). This causes 3d^9^-like electronic configurations for the Cu active site to be favored over reference 3d^10^ configurations at several points in the NO reaction cycle, giving rise to self-consistent PZSIC densities that are qualitatively incorrect. By locally scaling the SIC, LSIC mitigates some of the PZSIC errors and gives more consistent results than PZSIC-LDA. We conclude that to obtain accurate descriptions of catalytic reactions with SIC-based methods, the spurious energetics of transition metal complexes in competing oxidation states must be eliminated.

## Graphical abstract

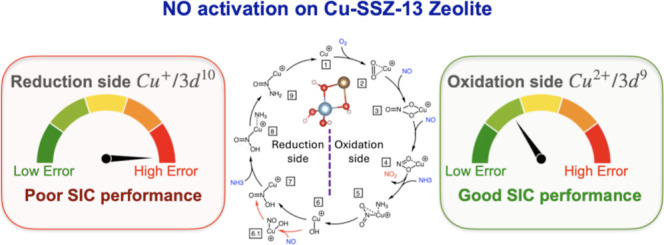

## 1 Introduction

The self-interaction error (SIE) in density functional theory (DFT) leads to inaccurate predictions of important properties such as chemical reaction barriers, electron removal energies, dissociation energies, and others.^1–7^ One-electron SIE arises from the incomplete cancellation of the self-Coulomb and self-exchange-correlation energies of electrons in approximate density functionals. For chemical reaction barriers, DFT tends to underpredict the energy of the transition state^3,4,8,9^ relative to the reactant or product, making the barriers too small. This underestimation results from stretched bonds in the transition state^3,10,11^ that correspond to orbitals that are delocalized over multiple atoms.

The Perdew–Zunger self-interaction correction (PZSIC) method^12^ removes SIE on an orbital-by-orbital basis, making any density functional approximation (DFA) exact in the limit of a one-electron density. PZSIC can significantly improve the description of reaction barriers^3,13,14^ by correcting the error associated with stretched bonds, raising the total energy of the transition state relative to that of the reactants and products. However, the relative energies of molecules with atoms near their equilibrium positions, e.g., in the reactants and products, can be made worse by PZSIC, and this limits the accuracy of barriers computed using PZSIC. This paradoxical behavior-improving the description of stretched bonds while degrading that of near-equilibrium bonds-was explained in part by Santra and Perdew,^15^ who showed that PZSIC introduces significant errors in the exchange–correlation energy in the uniform electron density limit, where standard DFT methods are exact by construction. In this work, we also employ the locally scaled self-interaction correction (LSIC) method developed by Zope et al.^16^ In this approach, a suitably chosen weighting function scales down the self-interaction correction in regions where the electron density varies slowly, while retaining the full correction in regions where the density is effectively one-electron-like. In their original work, Zope et al.^16^ used *z*_σ_, the ratio of von Weizsäcker and Kohn–Sham kinetic energy densities, as the weighting function to scale the SIC energy densities. With this choice, LSIC­(*z*_σ_) is exact in both the one-electron limit and the uniform electron gas limit, provided that the underlying density functional is exact in the latter. Weighting factors other than *z*_σ_ have been used in the LSIC scheme^17,18^ and are discussed later in the text. All LSIC calculations reported here use LSIC­(*z*_σ_). They are also based on the local density approximation (LDA) as the underlying DFA. It has been shown that the local scaling approach results in gauge inconsistencies when used with generalized gradient approximations (GGAs) and meta-GGAs and is less successful in those cases.^19^ For the sake of brevity, we will refer to LSIC­(*z*_σ_)-LDA in the remainder of the paper simply as LSIC.

LSIC improves the description of near-equilibrium properties such as atomization energies, ionization energies, polarizabilities, electron affinities, and spin-state gaps compared to PZSIC.^20–25^ It also reduces the delocalization errors substantially.^26^ Mishra et al.^14^ demonstrated that while PZSIC and LSIC both significantly improve reaction barrier predictions for the BH76 gas-phase benchmark data set, LSIC gives smaller errors, achieving a mean absolute error of only 2 kcal/mol for hydrogen transfer reactions.

Recently, some of us investigated a positive SIC energy shift for the localized orbitals in transition metal atoms in PZSIC calculations, due to their noded or lobed character.^27^ The shift is greatest when the 3d shell is completely filled, leading to a relative destabilization of a 3d^10^ configuration compared to 3d^9^ which manifested as an underestimation of computed PZSIC ionization energies. The underestimation is mitigated in corresponding LSIC calculations.^27^ Since many catalytic reactions involve a change in the oxidation state of a transition metal atom during the reaction, corresponding to a change in the occupation of the d orbitals, it is important to understand whether spurious effects analogous to the underestimated ionization energies impact the description of transition metal-based catalytic reactions using SIC-based methods.

In this work, we consider the selective catalytic reduction of NO over the copper-exchanged zeolite Cu-SSZ-13. This reaction has been extensively studied with a variety of methods due to its high activity and stability under reaction conditions.^28–43^ To assess the SIE problem affecting DFT treatments of this reaction, Paolucci et al.^44,45^ used the hybrid HSE06 functional to achieve better agreement with experimentally measured barriers for NO-assisted NH_3_ activation. Chen et al.^46^ applied PBE + U with entropic corrections to obtain accurate free-energy barriers. In addition, Goncalves et al.^47,48^ used DLPNO–CCSD­(T) to benchmark reaction energies and showed that standard GGA functionals can incur mean absolute errors exceeding 40 kJ/mol. These studies collectively highlight the need for improved functionals that reduce SIE.^44,49–60^

Motivated by these findings, we present the first comprehensive assessment of whether the SI-corrected functionals FLOSIC-LDA and LSIC-LDA improve DFT predictions of reaction energetics in Cu-zeolite catalysis. Specifically, we compare the performance of LDA, PBE, r^2^SCAN, and SI-corrected PZSIC-LDA and LSIC for computing the reaction barriers involved in the NO activation cycle over Cu-SSZ-13, using the Cu-SSZ-13 T1 cluster model (Figure 1) introduced by Goncalves et al.^47^ The overall stoichiometry of the reaction cycle is

$$2{NH}_{3}+3NO+O_{2}->N_{2}+3{H\;}_{2}O+{NO}_{2}$$ 1

**Figure 1:**
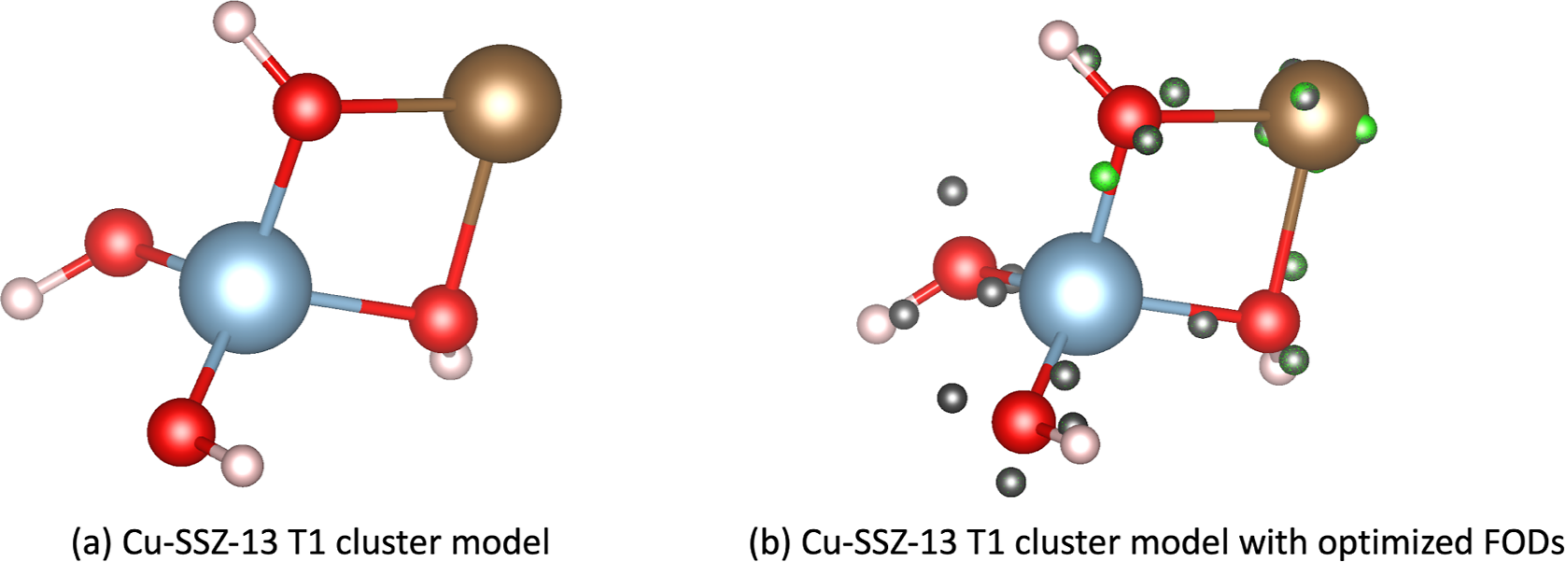
(a) Cu-SSZ-13 zeolite T1 cluster model. (b) Cu-SSZ-13 zeolite T1 cluster model with optimized up-spin and down-spin FOD positions. Color scheme: Cu (brown), Al (silver), O (red), H (pink), up-spin FOD (green), down-spin FOD (black).

The cycle comprises nine steps originally proposed by Janssens et al.^53^ While previous studies^47,48^ focused on only three of the nine steps, we compute reaction energies and barriers for all nine steps, comparing both DFT and SIC-based methods to reference CCSD­(T) results.

The NO activation cycle is an ideal context for testing SIC methods because it involves a multistep redox mechanism that allows us to investigate whether PZSIC and/or LSIC can offer a balanced and reliable description of both transition states and equilibrium states in catalytic systems where SIE is known to be significant and where the oxidation state of the Cu catalyst changes.

## 2 Theory and Computational Methods

### 2.1 FLOSIC Formulation

Any approximate DFT total energy, *E* ^DFT^[*n*_↑_, *n*_↓_], can be made free of one-electron SIE by applying the PZSIC correction.^12^ The PZSIC total energy is expressed as

2 $$E^{PZSIC}\left[n_{↑},n_{↓}\right]=E^{DFT}\left[n_{↑},n_{↓}\right]-\sum_{i,\sigma }\left(U\left[n_{i\sigma }\right]+E_{XC}^{DFT}\left[n_{i\sigma },0\right]\right)$$

where *U*[*n*_*i*σ_] and *E*_XC_^DFT^[*n*_*i*σ_,0] represent the self-Coulomb and self-exchange correlation energies, respectively, evaluated using the single-orbital densities *n*_*i*σ_(*r*). Because *E* ^PZSIC^ is orbital dependent, it matters which orbital densities are used in eq 2. We use the Fermi-Löwdin Orbital Self-interaction correction (FLOSIC) method^61–64^ to obtain localized orbitals that minimize the PZSIC total energy. In this approach, an orthonormal set of Fermi-Löwdin orbitals (FLOs) are constructed via a unitary transformation of the canonical Kohn–Sham orbitals. This transformation is governed by a set of parameters known as Fermi orbital descriptors (FODs) that are *N* points (for *N* electrons) in the space occupied by the electrons. Derivatives of the total energy with respect to the FODs can be computed,^62^ so that gradient-based methods can be used to determine the FODs, and hence FLOs, that minimize the PZSIC total energy. We used multiple starting points for the FOD searches. In a few cases, this led to a metastable arrangement of the FODs, as well as the lowest-energy arrangement, as discussed further below. In Figure 1, we show the T1 cluster model of Cu-SSZ-13 and its optimal set of FODs. Additional details on the construction and optimization of FODs and FLOs can be found in previous publications.^11,14,62,65^ In the text below, we refer to PZSIC calculations performed using the FLOSIC approach as FLOSIC-DFT, or more simply, FLOSIC calculations.

We point out that *E* ^*DFT*^ is a part of the PZSIC total energy. In a self-consistent FLOSIC calculation, the FLOSIC density is used to evaluate *E* ^DFT^ instead of the self-consistent DFT density. We dub this the DFT@FLOSIC energy and note that it can give insight into the difference between the DFT and FLOSIC-DFT densities and how that relates to SIE.^14^ The remainder of the total energy in eq 2 is the SIC part.

### 2.2 Locally Scaled SIC (LSIC­(*z*_σ_))

The LSIC method, developed by Zope et al.,^16^ is based on locally scaling the SIC to remove the correction where the density is slowly varying and apply it at full strength where the density is one-electron-like. It employs eq 2 with the following modifications

$$U^{LSIC}\left[n_{i\sigma }\right]=\frac{1}{2}\int d^{3}rz_{\sigma }\left(\mathrm{r⃗}\right)n_{i\sigma }\left(\mathrm{r⃗}\right)\int d^{3}\;\,{rz}^{\prime }\frac{n_{i\sigma }\left(\overset{⃗}{r^{\prime }\;}\right)}{\left|\mathrm{r⃗}-\overset{⃗}{r^{\prime }\;}\right|}$$ 3

and

$$E_{XC}^{LSIC}\left[n_{i\sigma },0\right]=\int d^{3}\;rz_{\sigma }\left(\mathrm{r⃗}\right)n_{i\sigma }\left(\mathrm{r⃗}\right)\varepsilon_{XC}^{DFT}\left(\left[n_{i\sigma },0\right],\mathrm{r⃗}\right)$$ 4

where ε_XC_^DFA^ denotes the DFT exchange–correlation energy density. In these expressions, $z_{\sigma }\left(\mathrm{r⃗}\right)=\frac{\tau_{\sigma }^{W}\left(\mathrm{r⃗}\right)}{\tau_{\sigma }\left(\mathrm{r⃗}\right)}$ is a position dependent scaling factor that ranges from zero for a uniform electron density to one for a one-electron density. Here, τ_σ_ is the positive kinetic energy density for occupied orbitals of spin σ and $\tau_{\sigma }^{W}=\frac{|\nabla n_{\sigma }{|}^{2}}{8n_{\sigma }}$ is the von Weizsäcker kinetic energy density. τ_σ_^*W*^ = τ_σ_ for a single-electron density. In our study, we evaluate *E* ^LSIC^ energies on self-consistent FLOSIC-LDA densities. Recent work has shown that such “one-shot” LSIC results closely resemble those obtained using fully self-consistent LSIC calculations.^66^

### 2.3 Computational Details

Structures for the T1 cluster model and the reactants and products of nine reactions were obtained from Goncalves et al.^47^ These structures were then reoptimized at the PBE level of theory^67^ with the def2-TZVPP basis set using the ORCA package.^68^ Atom-pairwise dispersion corrections were included using the Becke-Johnson damping scheme (D3BJ).^69,70^ Transition states for both single- and multistep reactions were located using the climbing image nudged elastic band (CI-NEB) method^71,72^ as implemented in ORCA. Single-point energy evaluations were carried out on the optimized structures at the CCSD­(T)/def2-TZVPP level of theory, which has been used previously as a benchmark in similar studies.^47,48^

DFT, FLOSIC-LDA, and LSIC calculations were performed using the FLOSIC code,^73^ which is based on the earlier NRLMOL code,^74,75^ using extensive Cartesian Gaussian basis sets.^76^ For example, the Cu basis used in these calculations features 20 Gaussian exponents configured into 7 *s*-type, 5 *p*-type, and 4 d-type functions. A discussion of the performance of NRLMOL basis sets can be found in ref 23. DFT total energies were computed with the local density approximation (LDA) in the Perdew–Wang parametrization (LDA-PW91),^77^ the Perdew–Burke–Ernzerhof (PBE)^67^ generalized gradient approximation (GGA), and the regularized and restored strongly constrained and appropriately normed (r^2^SCAN)^78^ meta-GGA functional. FOD positions were optimized via the L-BFGS algorithm^79^ until the maximum force on any FOD fell below 10^–3^ Ha/Bohr. The self-consistent field (SCF) convergence criterion was set to 10^–6^ Ha. All calculations were spin-unrestricted regardless of the expected spin state of a particular configuration.

## 3 Results and Discussion

We investigate the reaction energetics associated with the complete NO activation cycle (eq 1) on the Cu-SSZ-13 T1 zeolite cluster model, shown in Figure 1. Energies are reported in kJ/mol throughout this work. For reference, 1 kJ/mol = 0.238 kcal/mol = 0.0104 eV. The model features an Al atom with a nominal negative charge that is compensated by a positively charged Cu atom in the Cu­(I) oxidation state (3*d* ^10^). The reaction scheme for the NO activation cycle, originally proposed by Janssens et al.,^53^ is illustrated in black in Figure 2. We identified transition states and reaction pathways for both single- and multistep processes.

**Figure 2:**
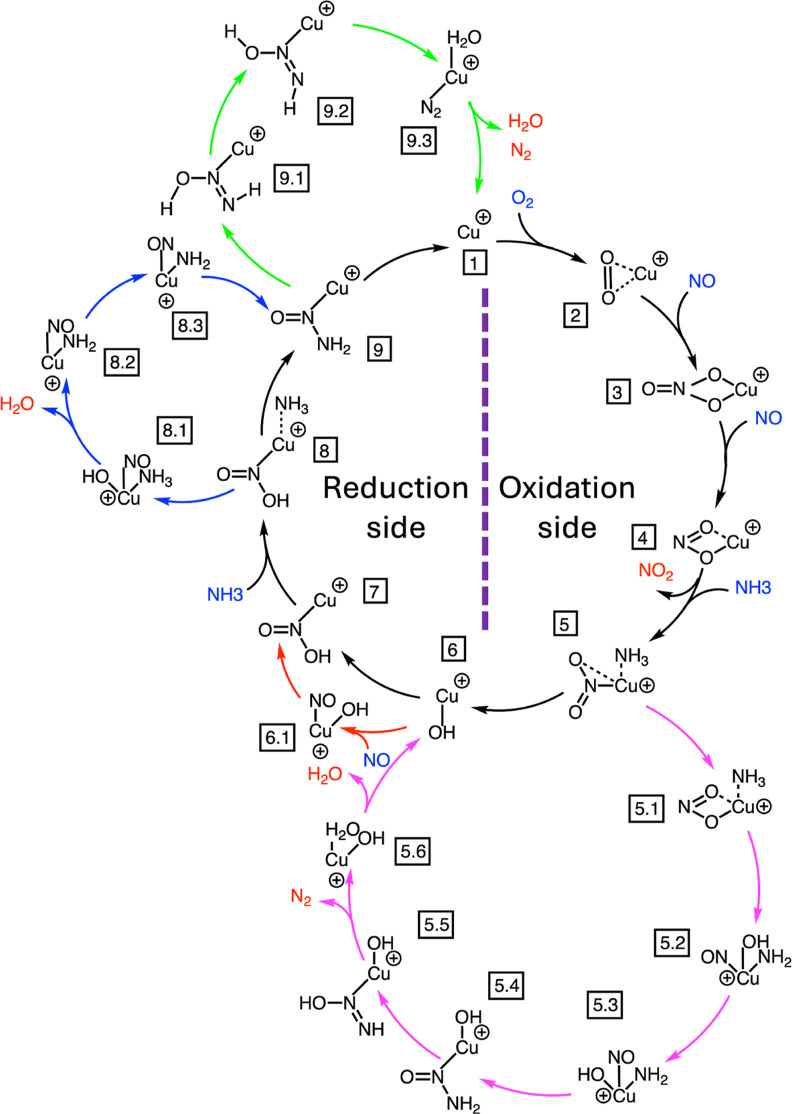
Overall NO activation cycle is represented in black. The active zeolite cluster (structure 1) is represented as “Cu^⊕^”. The entering gases such as O_2_, NO, NH_3_ are highlighted in blue whereas the leaving gases such as N_2_, H_2_O, and NO_2_ are highlighted in red. The multistep reactions structure 5 → structure 6, structure 8 → structure 9, structure 9 → structure 1 are highlighted in magenta, blue, and green, respectively. Structures 2*, TS­(2*-3), 5.2, TS(5.2–5.3), and 5.3 show strong multireference characters (T1 diagnostic ≥0.05; see Table S1), making CCSD­(T) energies for these structures potentially unreliable. Thus, we exclude reactions 2 → 3 and 5 → 5.4 from the analysis reported in Tables 2 and 4

We limited our study of SIC methods to FLOSIC-LDA and LSIC. Comparing FLOSIC-LDA results against uncorrected DFT results should suffice to identify where SIE plays a significant role. In addition, comparing LSIC and FLOSIC-LDA should identify cases where FLOSIC falters by overcorrecting LDA energies. Also, LSIC has shown comparable or superior performance to FLOSIC-PBE and FLOSIC-SCAN on the BH76 reaction set,^80^ suggesting that LSIC results can indicate how well other FLOSIC-based methods might perform for the reactions in the NO cycle. Finally, in our previous work on TM ionization energies,^27^ LSIC reduced the spurious destabilization of 3d^10^ configurations relative to 3d^9^, making it important to test whether it lessens problems related to destabilization of the Cu­(I) oxidation state relative to Cu­(II) (3d^9^) over the NO cycle.

### 3.1 Overview of Reaction Pathways

The NO activation cycle is shown in Figure 2. The nine steps are as follows: (i) adsorption of O_2_ from the gas phase onto the Cu T1 zeolite cluster (structure 1) to form Cu–O_2_ (structure 2); (ii) reaction of NO gas with Cu–O_2_ to form Cu–NO_3_ (structure 3); (iii) Cu–NO_3_ reacts with NO to form Cu-NO_2_ (structure 4) and NO_2_ gas; (iv) adsorption of NH_3_ onto Cu-NO_2_ to form Cu–NO_2_NH_3_ (structure 5); (v) Cu–NO_2_NH_3_ decomposes to Cu–OH (structure 6), N_2_, and H_2_O; (vi) reaction of NO with Cu–OH to form Cu–HONO (structure 7); (vii) adsorption of NH_3_ onto Cu–HONO to form Cu–NOOHNH_3_ (structure 8); (viii) Cu–NOOHNH_3_ decomposes to form Cu–NONH_2_ (structure 9) and H_2_O; (ix) Cu–NONH_2_ decomposes to regenerate structure 1, releasing N_2_, and H_2_O. Steps (i), (iv), and (vii) are direct adsorption steps, while (ii) are (iii) are single-step and (v), (vi), (viii) and (ix) are multistep reactions. The multistep reactions are described further below.

Step (v) begins with an NO_2_ molecule that is N-bound to structure 5. This species rearranges into structure 5.1, where the NO_2_ molecule adopts an O, O′-bound nitrito configuration, forming Cu–ONONH_3_. In structure 5.2, a hydrogen transfers from the NH_3_ molecule to the NO_2_ group, generating adsorbed NH_2_, OH, and NO molecules. These adsorbates undergo rearrangement in structure 5.3. Structure 5.4 involves N–N bond formation, yielding adsorbed OH and ONNH_2_ molecules. A subsequent hydrogen transfer leads to structure 5.5, with an adsorbed HNNOH intermediate. N_2_ is released, leaving behind adsorbed OH and H_2_O in structure 5.6. In the final step, H_2_O desorbs, resulting in structure 6, a Cu–OH complex.

Step (vi) proceeds via two elementary steps: (i) adsorption of NO onto Cu–OH to form coadsorbed OH and NO groups on the Cu-atom (structure 6.1); and (ii) formation of the Cu–HONO complex (structure 7).

In Step (viii) the N–O bond in the Cu–NOOHNH_3_ complex (structure 8) breaks to form adsorbed NO, OH, and NH_3_ molecules on Cu, resulting in structure 8.1. Subsequently, a hydrogen atom transfers from the NH_3_ molecule to form H_2_O, which binds via van der Waals (vdW) interactions with adsorbed NO and NH_2_ molecules on Cu in structure 8.2. Next, H_2_O is removed, and the Cu-complex relaxes with the remaining bound NO and OH groups, yielding structure 8.3. This intermediate further relaxes to form Cu–ONNH_2_ (structure 9).

Step (ix) involves the decomposition of the ONNH_2_ molecule. This has been studied previously and the key intermediates are well established.^81–84^ First, a hydrogen atom transfers from the NH_2_ group to the oxygen atom in Cu–ONNH_2_ (structure 9), forming Cu–HN=NOH (structure 9.1). The next step (structure 9.1 → 9.2) involves rotation of the two hydrogen atoms. Finally, in the third step, a hydrogen atom transfers from the NH group to the OH group, forming N_2_ and H_2_O molecules adsorbed on the Cu cluster (structure 9.3). In the final step of the cycle, both H_2_O and N_2_ detach into the gas phase, regenerating the bare Cu cluster (structure 1).

The T1 diagnostic^85^ was used to check all single point CCSD­(T) calculations for multireference character. Only the transition state between structures 2 and 3, the transition state between structures 5.2 and 5.3, and structure 5.3 were found to have a T1 value greater than the threshold 0.05, whichindicates significant multireference character and potentially unreliable energies for these structures.^85^ We therefore omit reaction barriers for reactions 2 → 3 and all reactions from 5 → 5.4 from our analysis below. None of the structures on the reduced side of the reaction has a T1 diagnostic value that exceeds the threshold. See Table S1 for a full listing of T1 values for all calculations.

The CCSD­(T) Mulliken charge of Cu in structure 1, where Cu is expected to be in the Cu­(I) oxidation state, is +0.65*e*. In structure 2, the Cu Mulliken charge is +1.06*e*. We take this larger value to indicate that Cu in structure 2 is in the Cu­(II) oxidation state. The Cu Mulliken charges in all other structures lie close to one or the other of these values and we assume that they are indicative of Cu­(I)/Cu­(II) oxidation states (Figure S1). Using this approach, adsorption of O_2_ in reaction step (i) changes the oxidation state from Cu­(I) to Cu­(II). Cu then remains as Cu­(II) from structure 2 to structure 6, referred to below as the oxidized side of the overall reaction cycle. Cu­(II) is reduced to Cu­(I) in structure 6*, which is a vdW complex of structure 6 and NO, and remains Cu­(I) through the completion of the cycle, which we refer to as the reduced side of the cycle.

For comparison, the Mulliken charge of Cu in structure 1 is 0.8*e* in the FLOSIC-LDA calculation, changing to about 1.4*e* in structure 2 (See Figure S2). This is consistent with the Cu­(I) to Cu­(II) change predicted by CCSD­(T). FLOSIC-LDA Mulliken charges for Cu remain near 1.4*e* for all structures on the oxidized side of the reaction, again consistent with CCSD­(T) in predicting Cu­(II) charge states. On the reduced side, however, the FLOSIC-LDA Mulliken values for Cu remain near 1.4*e* for the ground state configurations, while in some cases the values are close to 0.8*e* for metastable states. Thus, FLOSIC-LDA appears to incorrectly predict Cu­(I) for the reduced side of the reaction.

By contrast, Mulliken charges for Cu for structures 1 and 2 are approximately 0.3*e* and 0.4*e*, respectively, in the PBE calculations (Figure S3). The smaller difference makes identifying Cu charge states difficult in the PBE case. The Cu Mulliken charges on the oxidized side of the cycle for PBE show more variability than for either CCSD­(T) or FLOSIC-LDA and several structures on the oxidized side have Cu Mulliken charges less than 0.3*e*. On the reduced side, the Cu Mulliken charges are more consistent, with all values at or smaller than 0.3*e*, suggesting Cu­(I) charge states in all cases, in agreement with the reference CCSD­(T) calculations.

### 3.2 Performance of Uncorrected DFT Methods

We begin by assessing the performance of DFT (LDA, PBE, and r^2^SCAN) functionals in describing the adsorption energies and reaction barriers involved in the NO reaction cycle. These DFAs increase in sophistication and expected accuracy in the order LDA < PBE < r^2^SCAN. Other evaluations of DFT performance for catalytic reactions have appeared recently.^86^

Adsorption steps in the NO cycle occur at (i), (iv), (vi), and (vii). In Table 1, we compare the calculated DFT adsorption energies for these steps with the CCSD­(T) reference values. In essentially all cases, the DFT methods predict significantly stronger adsorption than CCSD­(T), in some cases for LDA by more than 100 kJ/mol. The r^2^SCAN results improve over LDA and PBE relative to CCSD­(T) values in most cases. The MAE is slightly smaller for r^2^SCAN (44.29 kJ/mol) than PBE (44.61 kJ/mol) and both are much smaller than for LDA (83.63 kJ/mol).

**Table 1:** Adsorption Energies (*E*(*P*)–*E*(*R*)) for Reaction Steps 1 → 2, 4 → 5, 6 → 6.1, 7 → 8 (in kJ/mol) Computed with LDA, PBE, r^2^SCAN, and CCSD­(T)^a^

	LDA	PBE	r^2^SCAN	CCSD(T)
1 + O_2_(g) → 2	–168.24	–122.63	–144.54	–65.16
4 + NH_3_(g) → 5	–124.27	–77.19	–69.98	–37.42
6 + NO(g) → 6.1	–172.12	–114.01	–106.91	–44.44
7 + NH_3_(g) → 8	–106.39	–65.87	–74.74	–77.50
ME	–83.63	–38.80	–42.91	
MAE	83.63	44.61	44.29	

a Mean errors (ME) and mean absolute errors (MAE) are relative to CCSD­(T) values.

The remaining steps of the cycle involve reactions with energy barriers separating the reactants (R) and products (P) from the transition states (TS). In Table 2, Vf represents the forward barrier (reactants vs TS; *E*(*TS*)–*E*(*R*)) and Vr the reverse barrier (products vs TS; *E*(*TS*)–*E*(*P*)) for these reactions. The table shows mean (ME) and mean absolute (MAE) errors in barriers relative to CCSD­(T) values. The results are separated for those on the oxidized and reduced sides of the reaction. Complete results for each method at each reaction step are provided in Tables S2 and S3 in the Supporting Information.

**Table 2:** Mean Absolute Errors (MAE) in Forward (*V*_f_; *E*(*TS*) – *E*(*R*)) and Reverse (*V*r; *E*(TS) – *E*(*P*)) Reaction Barriers and Reaction energies (rxn; *V*_f_–*V*_r_) Relative to CCSD­(T) energies (in kJ/mol) for LDA, PBE, and r^2^SCAN for Reactions on the Oxidized Side (3 → 4, 5.4 → 5.5, 5.5 → 5.6) and Reduced Side (6.1 → 7, 8 → 8.1, 8.1 → 8.2, 8.3 → 9, 9 → 9.1, 9.1 → 9.2, 9.2 → 9.3) of the NO Reaction Cycle^a^

		LDA	PBE	r^2^SCAN
Oxidized	*V*_f_	45.43	38.22	28.31
	*V*_r_	87.02	61.44	45.24
	rxn	64.04	44.78	30.15
Reduced	*V*_f_	16.85	15.34	12.90
	*V*_r_	30.14	26.38	20.21
	rxn	16.13	15.01	14.73
all barriers		36.32	29.55	22.62

a The MAE including all forward and reverse barriers for the full cycle is also shown.

The DFT methods nearly always underestimate the reference CCSD­(T) barrier heights. The MAE decreases from LDA to PBE to r^2^SCAN for both forward and reverse barriers, and for the oxidized and reduced sides of the cycle. The MAE for all methods are smaller on the reduced side. This is likely because the average barrier height on the reduced side (108.39 kJ/mol) is roughly half that on the oxidized side (195.18 kJ/mol).

Table 2 also shows how DFT reaction energies, computed as *V*_f_ – *V*_r_ for a given reaction, compared with CCSD­(T) values. Again the MAE decreases from LDA to PBE to r^2^SCAN, and again the MAE on the reduced side of the reaction are smaller than on the oxidized side.

### 3.3 Performance of FLOSIC Methods

In this section, we present results for LDA@FLOSIC, FLOSIC-LDA and LSIC methods for the NO reaction cycle. In the first subsection, we briefly describe the recently identified SIC energy penalty^27^ associated with the lobedness of local orbitals and discuss how it could impact SIC-based results for the NO cycle. We then present results for the oxidized side of the cycle, followed by results for the reduced side. Discussion of all SIC-based results follows.

#### 3.3.1 The PZSIC Energy Penalty

We recently documented^27^ an effective SIC energy penalty in FLOSIC-DFT total energies for atoms involving noded 3d orbitals. The penalty stems from the fact that DFAs overestimate the magnitude of the exchange energy when evaluated on noded or lobed densities.^8^ The orbital-by-orbital subtraction of negative self-exchange-correlation energies in the PZSIC energy (eq 2) then results in a positive shift of the SIC part of the energy. Although this shift is present for all orbitals, it is larger when the orbitals are more lobed. For the 3d transition metal atoms, it is largest for orbitals belonging to 3d^5^ or 3d^10^ electronic configurations where the FLOs are most lobed. The difference in the size of the shift for a 3d^10^ compared to a 3d^9^ configuration can be significant and is linked to a severe underestimation of corresponding FLOSIC-DFT ionization energies compared to experimental values.^27^ For example, the ionization of Cu^+^ (3d^10^) to Cu^2+^ (3d^9^) is overestimated by LDA by 1.51 eV, but underestimated by FLOSIC-LDA by 2.02 eV.^27^ The SIC contribution to the FLOSIC-LSDA ionization energy is approximately the difference in these values, about −3.5 eV or −340 kJ/mol. LSIC significantly reduces the FLOSIC-LDA error to −0.37 eV (−36 kJ/mol).

The energy penalty can be viewed as a destabilization of a 3d^10^ vs a 3d^9^ electronic configuration. This could affect the FLOSIC results for the NO cycle in two ways. First, when a reaction step involves a change in oxidation state, the destabilization of Cu­(I) relative to Cu­(II) could cause an error in reaction energies. Going from Cu­(I) to Cu­(II), the error would be negative and the FLOSIC reaction energy would be too exothermic. Conversely, for a reaction that goes from Cu­(II) to Cu­(I), the error would be in the opposite direction, making the reaction too endothermic. By analogy to the ionization energies in ref 27, LSIC might be expected to decrease this type of error by scaling down the size of the SIC part of the energy.

The above analysis assumes that FLOSIC predicts the correct oxidation state for all species involved in a reaction, despite the relative destabilization of Cu­(I). A second type of error would occur if FLOSIC incorrectly predicts a Cu­(II) oxidation state instead of Cu­(I) because of the destabilization. This would result in qualitatively incorrect charge densities and hence unphysical descriptions and unreliable energies of the complexes involved for all SIC-related methods. As described above, FLOSIC-LDA calculations, based on Cu Mulliken charges, predict a Cu­(II) ground state for all complexes on the reduced side of the cycle, whereas the reference CCSD­(T) calculations indicate Cu­(I). As shown in Figure S2, metastable Cu­(I) solutions are found for some of the species on the reduced side. In particular, we find both Cu­(I) and Cu­(II) solutions for all species involved in the reaction step 9.2 → 9.3. We compare and contrast descriptions of this reaction based on the two solutions in Section 3.3.3.

#### 3.3.2 FLOSIC-DFT for the Oxidized Side of the Cycle

In Table 3, we show calculated adsorption energies for the three adsorption events on the oxidized side of the NO cycle. Results for LDA, LDA@FLOSIC, FLOSIC-LDA, and LSIC are presented, along with the reference CCSD­(T) values.

**Table 3:** Adsorption Energies, (*E*(*P*) – *E*(*R*)), for Reactions 1 → 2, 4 → 5, and 6 + NO­(g) → 6* (in kJ/mol) Computed with LDA, LDA@FLOSIC, FLOSIC, LSIC­(*z*_σ_), and CCSD­(T) Energies, Along With Mean Absolute Error (MAE), and Mean Error (ME) (in kJ/mol)^a^

		LDA	LDA@FLOSIC	FLOSIC	LSIC(*z*_σ_)	CCSD(T)
1 + O2(g) → 2	3d^10^→ 3d^9^	–168.24	–53.40	–376.57	–82.26	–65.16
4 + NH3(g) → 5	3d^9^ → 3d^9^	–124.27	–63.56	–72.79	13.76	–37.42
6 + NO(g) → 6*	3d^9^ → 3d^10^	–121.51	–73.73	326.76	147.61	–5.54
	3d^9^ → 3d^9^		71.66	68.36	143.51	
ME		–101.97	–27.52	–4.83	62.41	
MAE		101.97	35.37	226.40	73.81	

a Here, the Cu ion is in the Cu­(I) oxidation state (3d^10^) in structures 1 and 6* and in Cu­(II) (3d^9^) in the remaining structures.

The first reaction involves structures 1 and 2 in Cu­(I) and Cu­(II) oxidation states, respectively. O_2_ is relatively strongly adsorbed in CCSD­(T) with adsorption energy −65.16 kJ/mol. LDA strongly overbinds the O_2_ molecule by an additional 103.08 kJ/mol relative to CCSD­(T). LDA@FLOSIC performs much better than LDA, underbinding the CCSD­(T) result by only 11.76 kJ/mol. FLOSIC-LDA, on the other hand, significantly overbinds it, by 311.51 kJ/mol. Finally, LSIC corrects much of the FLOSIC-LDA error, overbinding O_2_ by only 17.10 kJ/mol, relative to the reference.

In step (iv), NH_3_ binds to structure 4, with a CCSD­(T) adsorption energy of −37.42 kJ/mol. Compared to this, LDA, LDA@FLOSIC, and FLOSIC-LDA overbind the NH_3_ molecule by 86.85, 26.15, and 35.38 kJ/mol, respectively. LSIC significantly underbinds NH_3_ by 51.18 kJ/mol relative to CCSD­(T), predicting the complex to be unbound by 13.76 kJ/mol. This may reflect a problem describing weak bonds involving closed-shell molecules that has been attributed to LSIC.^18,87^

Lastly, in the reaction 6 + NO­(g) → 6*, NO adsorbs weakly to structure 6, with CCSD­(T) adsorption energy of −5.54 kJ/mol. LDA strongly overbinds NO, by 115.97 kJ/mol. In Table 3, we show two sets of FLOSIC-LDA results for this reaction. The first uses the metastable, but qualitatively correct, 3d^10^ solution for the 6* complex. For this set, LDA@FLOSIC significantly improves on the LDA overbinding, but FLOSIC-LDA makes a dramatic error of 332.30 kJ/mol in the adsorption energy, leaving NO strongly unbound. Here Cu is reduced from Cu­(II) in the reactant to Cu­(I) in the product and the FLOSIC-LDA error is reversed in sign from the large error for reaction 1. Again LSIC reduces the size of the FLOSIC-LDA error, but still leaves NO unbound by 147.61 kJ/mol.

The second set of results uses the ground state 3d^9^ solution for 6*. For this set, LDA@FLOSIC makes a dramatic error in the adsorption energy, leaving NO unbound by 71.66 kJ/mol. FLOSIC-LDA makes an error similar to LDA@FLOSIC. This implies that the SIC part of the energy does not contribute to the error in this case. By contrast, LSIC makes a much larger error, leaving NO unbound by over 140 kJ/mol.

The ME and MAE for the adsorptions are also shown in Table 3. Only the results for the 3d^9^ → 3d^10^ FLOSIC-LDA solution for the third reaction are used to compute these averages. FLOSIC-LDA gives a very small ME of −4.83 kJ/mol, but this reflects a strong cancellation of errors. LDA@FLOSIC also has a relatively small ME of −27.52 kJ/mol, which is smaller than either PBE or r^2^SCAN (−55.77 and −55.51 kJ/mol, respectively). The ME for LSIC is positive and much larger, 62.41 kJ/mol. The MAE for LDA@FLOSIC remains relatively small, 35.37 kJ/mol, but is much larger for FLOSIC-LDA (226.40 kJ/mol) and LSIC (73.81 kJ/mol). The large FLOSIC-LDA MAE is because of the large errors for the first and third adsorptions where the Cu-oxidation state changes. LSIC reduces, but does not eliminate, the FLOSIC-LDA errors in these cases.

In Table 4, we present results for forward (*V*_f_) and reverse (*V*_r_) reaction barriers for the oxidized side of the NO cycle. In all of these reactions, the lowest-energy FLOSIC-LDA solutions predict the qualitatively correct oxidation state (Cu­(II)) for the complexes involved.

**Table 4:** Individual Errors in Forward (*V*_f_; *E*(*TS*) – *E*(*R*)) and Reverse (*V*_r_; *E*(*TS*) – *E*(*P*)) Reaction Barriers Relative to CCSD­(T) energies, along with Mean Absolute Error (MAE), and Mean Error (ME) (in kJ/mol) for LDA, LDA@FLOSIC, FLOSIC-LDA (FLOSIC), and LSIC­(*z*σ) for Reactions 3 → 4 and 5.4 → 5.6

		LDA	LDA@FLOSIC	FLOSIC	LSIC(*z*_σ_)
step (iii)	*V*_f_ (3–4)	–89.16	–40.91	–38.57	38.76
	*V*_r_ (3–4)	–55.49	–94.09	–100.78	–23.69
step (v)	*V*_f_ (5.4–5.5)	–15.24	–1.69	15.03	11.80
	*V*_r_ (5.4–5.5)	–46.08	–34.33	52.11	31.30
	*V*_f_(5.5–5.6)	–31.89	59.28	137.51	23.44
	*V*_r_ (5.5–5.6)	–159.50	–31.99	10.88	14.14
	ME	–66.23	–23.95	12.70	15.96
	MAE	66.23	43.71	59.15	23.85

LDA underestimates all the reaction barriers listed in Table 4. The largest error, −159.50 kJ/mol, is for *V*_r_ (5.5–5.6). This step also has the largest CCSD­(T) barrier, 422.82 kJ/mol. LDA@FLOSIC improves the LDA barriers in four of the cases in Table 4. But for *V*_r_(3–4), it underestimates the CCSD­(T) barrier by −94.04 kJ/mol compared to −55.49 for LDA and for *V*_f_ (5.5–5.6), LDA@FLOSIC makes the barrier too large, overshooting the CCSD­(T) value by 59.28 kJ/mol. LDA underestimates here by −31.89 kJ/mol.

FLOSIC-LDA improves the LDA barriers in three cases in the table, but also worsens them in three. For *V*_r_ (3–4), the FLOSIC-LDA error (−100.78 kJ/mol) is nearly the same as LDA@FLOSIC (−94.09 kJ/mol), indicating that the SIC part of the energy contributes little to the error in this case. For *V*_r_ (5.4–5.5), FLOSIC-LDA overestimates the barrier by 52.11 kJ/mol, mostly due to a large positive SIC contribution. For *V*_f_ (5.5–5.6), FLOSIC-LDA strongly overestimates the barrier by 137.51 kJ/mol, again due to a large positive SIC contribution.

Finally, LSIC reduces LDA errors for all barriers in Table 4. The LSIC errors are seen to be similar in size for all barriers in the table, avoiding the large swings for FLOSIC-LDA.

The MAE for the barriers in Table 4 are smallest for LSIC, at 23.85 kJ/mol. The MAEs for LDA@FLOSIC (43.71) and FLOSIC-LDA (59.15) also improve over LDA(66.23), but less so than LSIC. Note that the ME for LDA@FLOSIC (−23.95) and FLOSIC-LDA (12.70) are relatively small, but both benefit from significant error cancellation.

#### 3.3.3 FLOSIC-DFT for the Reduced Side of the NO Cycle

As mentioned above, the ground state FLOSIC-LDA solutions predict a Cu­(II) oxidation state for all cluster complexes on the reduced side of the cycle, whereas the reference CCSD­(T) calculations indicate Cu­(I). The result for each individual reaction step is shown in Tables S2 and S3 in the Supporting Information for completeness, but because the FLOSIC-LDA calculations feature a physically incorrect electronic structure, we do not discuss the results for reduced side reactions here. However, for step 9.2 → 9.3, we found both 3d^9^ and metastable 3d^10^ solutions for all complexes involved in the reaction. Results for the barriers for this reaction are given in Table 5 for both the qualitatively incorrect FLOSIC-LDA ground states (3d^9^) and the correct metastable states (3d^10^).

**Table 5:** Errors in Reaction Barriers for the Reaction Step 9.2 → 9.3 Calculated using LDA, LDA@FLOSIC, FLOSIC, and LSIC­(*z*_σ_)­^a^

		LDA	LDA@FLOSIC	FLOSIC	LSIC(*z*_σ_)
3d^9^	Vf(9.2–9.3)		–29.66	–13.23	–27.21
	Vr(9.2–9.3)		–121.16	–159.67	–118.43
3d^10^	Vf(9.2–9.3)	–9.87	12.46	49.54	24.15
	Vr(9.2–9.3)	–83.19	–40.89	–45.15	–30.89

a For the FLOSIC-based calculations, results are shown for the lowest-energy, but qualitatively incorrect, solutions (3d^9^) for the reacting species, as well as for the qualitatively correct metastable solutions (3d^10^).

For the 3d^9^ FLOSIC solutions, LDA@FLOSIC, FLOSIC, and LSIC all underestimate both the forward and reverse barriers more than the corresponding LDA results. This means that the barriers are smaller in the FLOSIC-derived calculations than in the LDA calculations. For *V*_r_ (9.2–9.3); the LDA@FLOSIC (−121.16 kJ/mol) and FLOSIC-LDA (−159.67 kJ/mol) errors are especially large. The LSIC error is also large, at −118.43 kJ/mol.

For the 3d^10^ solutions the LDA@FLOSIC, FLOSIC, and LSIC barriers are larger than the LDA barriers. The barrier height errors are similar in size to the MAE shown in Table 4 for each method.

### 3.4 Discussion

Previous work showed that FLOSIC-DFT methods improve the description of both adsorption on transition metal sites^88^ and chemical reaction barrier heights^11,14,89^ compared to DFT calculations. The corrections come in part from changes in the self-consistent FLOSIC-DFT charge density that are captured in the DFT@FLO part of the energy. The remainder stem from the SIC part of the PZSIC total energy. The difference between the FLOSIC-DFT and DFT@FLO results is due to the SIC part of the energy. Similarly, the difference between the LSIC and DFT@FLO results reflects the scaled SIC energy.

FLOSIC-DFT methods were recently used to investigate the adsorption of seven small molecules on isolated closed-shell transition metal ions, including Cu^+^.^88^ It was argued that the results for these simple systems should be representative of results for adsorption on transition metal ions in the same charge states in more complex settings, like the T1 cluster model used here. The LDA adsorption energies of the molecules on a lone Cu^+^ were too large by −84 kJ/mol on average, similar to the average overbinding by LDA shown in Table 3 for O_2_, NH_3_, and NO, −102 kJ/mol. The overbinding on bare Cu^+^ was reduced to −56 kJ/mol for LDA@FLOSIC and −23 kJ/mol for FLOSIC-LDA in ref 88 (No LSIC results were reported in 88.) In Table 3, the LDA@FLOSIC results consistently improve over LDA, reducing the MAE from 101.97 to 35.37 kJ/mol, similar to the findings of ref 88. But the FLOSIC-LDA results in Table 3 are strikingly different, increasing the MAE to 226.4 kJ/mol due to the large errors for adsorption energies when the Cu-oxidation state changes. The magnitudes and signs of these errors are consistent with the expectations delineated in Section 3.3.1 that are based on the SIC energy penalty described in ref 27. We therefore conclude that the large errors seen in Table 3 reflect the destabilization of Cu­(I) relative to Cu­(II) due to the energy penalty. In Table S5, we present results for additional cases in which the oxidation state of the Cu changes. In all of these, the energy errors are consistent with the SIC energy penalty.

The present results for FLOSIC-LDA reaction barriers (Table 4) can be compared with previous FLOSIC-LDA calculations for reaction barrier heights.^11,14,89,90^ For the BH76 benchmark set, LDA calculations result in a MAE of 65 kJ/mol. This is reduced to 47 kJ/mol for LDA@FLOSIC, 23 kJ/mol for FLOSIC-LDA, and only 16 kJ/mol for LSIC.^14^ The MAE results in Table 4 are comparable to these for LDA (66 kJ/mol), LDA@FLOSIC (44 kJ/mol) and LSIC (24 kJ/mol), but, unlike the BH76 results, the MAE for FLOSIC-LDA (59.15 kJ/mol) in Table 4 is larger than LDA@FLOSIC. The implication is that the self-consistent FLOSIC-LDA density reduces errors as in the earlier BH76 calculations, but the SIC contribution to the total energy does not consistently improve the barriers further. For the reactions in Table 4, there is no change in the Cu-oxidation state, so this problematic performance of FLOSIC-LDA cannot be attributed to the SIC energy penalty. We note that the BH76 set does not include any systems containing transition metal atoms for comparison. The LSIC MAE (24 kJ/mol) is in line with the BH76 results. Scaling the SIC part of the PZSIC energy improves the description of the barriers in Table 4. We note that the MAE of LSIC in Table 4 improves over the average r^2^SCAN error for the same reactions, about 37 kJ/mol.

For completeness, for the reaction barriers of the BH6 reaction set, the MAEs are 76 kJ/mol for LDA, 40 kJ/mol for PBE, and 33 kJ/mol for r^2^SCAN.^89^ The ordering of the MAE in Table 2 is the same: LDA­(36.32 kJ/mol), PBE (29.55 kJ/mol) and r^2^SCAN (22.62 kJ/mol), obtained by averaging over the forward and reverse reactions for the oxidized and reduced sides of the cycle. The errors decrease with the increasing sophistication of the DFAs. The smaller values seen in Table 2 are likely due to smaller average barrier heights in the NO cycle compared to the BH6 set.

The results in Table 5 allow a comparison of FLOSIC-LDA barrier height calculations when correct and incorrect oxidation states are used in the calculations. When the qualitatively correct 3d^10^ oxidation state is used for Cu, the results are comparable to those in Table 4: LDA@FLOSIC increases the size of the LDA barriers, FLOSIC-LDA increases the barriers further, but does not improve over LDA@FLOSIC, and again LSIC improves over FLOSIC-LDA. For the 3d^9^ calculations the situation is very different. All FLOSIC-based methods worsen LDA’s underestimation of barriers, and the average LSIC error (72.82 kJ/mol) is much larger than for the reactions in Table 4. The use of qualitatively incorrect electron densities in these calculations leads to poor descriptions of reaction barriers.

## 4 Conclusion

In this work, we benchmarked the performance of standard DFT functionals LDA, PBE, and r^2^SCAN and the self-interaction-corrected DFT methods FLOSIC-LDA (which evaluates the PZSIC total energy^12^ using the LDA and the FLOSIC framework) and LSIC on the NO activation cycle that constitutes the selective catalytic reduction of NO_*x*_ over the Cu-SSZ-13 zeolite cluster model. The NO activation cycle consists of nine steps, including simple adsorptions at the Cu site (steps (i), (iv), and (vii)) and steps involving chemical reaction barriers. Our results reveal that the uncorrected DFT methods yield adsorption energies that are too negative (Table 1) and chemical reaction energies that are too small (Table 2). r^2^SCAN performs better than PBE and LDA in both cases. These results are consistent with those of prior studies and reflect the detrimental impact of SIE in describing chemical reactions. The success of FLOSIC-LDA and LSIC in remediating these errors is mixed. For example, LSIC improves the description of reaction barriers (Table 4) compared to r^2^SCAN, but not adsorption energies (Table 3).

The LSIC results presented here generally improve over FLOSIC-LDA. This may be due to the design of LSIC to reduce the magnitude of the PZSIC self-interaction correction in spatial regions where many orbitals overlap and the overall electron density varies slowly. This can account for LSIC being less susceptible to the SIC energy penalty by scaling down the SIC part of the energy. However, LSIC can give worse results than FLOSIC-LDA for weak bonds between molecules that involve overlapping tails of orbitals.^17,87^ LSIC using the *z*_σ_ scaling factor effectively turns off SIC in these regions, but gives essentially full correction in the corresponding orbital tail regions of the isolated molecules.^17^ The net effect is to destabilize the complex relative to the isolated molecules. This may explain why LSIC predicts a positive adsorption energy for NH_3_ at step (iv) of the reaction cycle, while CCSD­(T) predicts −37 kJ/mol. An updated approach to local scaling, LSIC-alpha,^18^ was recently developed to address this problem. The scaling factor in LSIC-alpha was designed to recognize overlapping orbital tail regions and to apply full SIC there. Initial tests^18^ show that the method performs significantly better than LSIC for weak bonds, although none of the tests involved transition metal complexes. It would be of interest to test LSIC-alpha for reactions like those studied here.

Perhaps the most significant finding of this work is that the SIC energy penalty that was identified earlier in the context of transition metal ionization energies^27^ and is due to the lobedness of the local orbitals used to evaluate the PZSIC total energy reappears in the present study as the destabilization of the Cu­(I) oxidation state relative to Cu­(II) for complexes involving the Cu-SSZ-13 cluster model. The penalty gives rise to large energy errors in the FLOSIC-LDA results for reaction steps that involve a change in the Cu-oxidation state (Tables 3 and S5). In addition, the penalty results in FLOSIC-LDA predicting (based on Mulliken charges for Cu) qualitatively incorrect Cu­(II) ground states for all complexes on the reduced side of the NO reaction cycle (Figure 2) where the reference CCSD­(T) calculations predict Cu­(I). Metastable Cu­(I) solutions are found in FLOSIC-LDA for some complexes on the reduced side. Focusing only on the reaction steps that are not affected by the SIC energy penalty, the FLOSIC-LDA and LSIC results presented here are consistent with the success of previous FLOSIC-based results for improving reaction barriers^11,14,89^ and molecular adsorption on a Cu site.^88^

While LSIC can reduce the size and impact of the SIC energy penalty,^27^ significant effects can still remain (compare the first and third lines in Table 3, for example). As discussed above, the source of the penalty is the lobedness of the orbitals used to evaluate the SIC portion of the FLOSIC energy. An alternative way to directly deal with orbital lobedness is by using complex local orbitals.^91,92^ It has been shown that using complex FLOs significantly reduces an energy penalty associated with the representation of double and triple bonds by lobed real FLOs.^93^ It would be interesting to investigate how fully the complex FLOSIC approach would eliminate the SIC energy penalty problem associated with changing oxidation states as in the NO reaction cycle. Recently, a self-consistent implementation of LSIC employing complex orbitals and the *z*_σ_ scaling factor was reported.^94^ Testing such an approach for reactions like those studied here would also be a promising direction for future research.

## Supplementary Material





## Notes

Tables for absolute forward and reverse barriers, errors (in kJ/mol) for LDA, LDA@FLOSIC, FLOSIC-LDA, LSIC­(*z*_σ_)-LDA, PBE, r^2^SCAN, errors in forward and reverse barriers with respect to CCSD­(T), difference between two FLOSIC-LDA energies for two local minima, errors in adsorption and reaction barriers for two FLOSIC-LDA solutions with respect to CCSD­(T) energies, and T1 diagnostic for all studied structures. Figures for the comparison of two FLOSIC-LDA solutions and Mulliken charges for studied structures (PDF)
Atomic coordinates and lowest-energy FOD structures for all systems (ZIP)
