# Supplementary material for: Assessing Self-Interaction Corrections in the Selective Catalytic Reduction of NO on a Cu-SSZ-13 Zeolite Cluster Model
Source: J Phys Chem A. 2026 Jul 20;130(30):5840–51. doi: 10.1021/acs.jpca.6c02902 (PMC13430625; doi:10.1021/acs.jpca.6c02902)
Supplement: Supplementary file 1 [file jp6c02902_si_001.pdf]

Supplementary Materials for: Assessing Self-Interaction Corrections in the Selective Catalytic Reduction of NO on a Cu-SSZ-13 Zeolite Cluster Model

Priyanka B. Shukla,[*,†] Selim Romero,[‡,‖] Tunna Baruah,[¶,‡] Rajendra R. Zope,[¶,‡] Koblar A. Jackson,[§] and J. Karl Johnson[†]

†*Department of Chemical & Petroleum Engineering, University of Pittsburgh, Pittsburgh, Pennsylvania 15261, United States*

‡*Computational Science Program, University of Texas at El Paso, El Paso, Texas 79968, United States*

¶*Department of Physics, University of Texas at El Paso, El Paso, Texas 79968, United States*

§*Physics Department and Science of Advanced Materials Program, Central Michigan University, Mount Pleasant, Michigan 48859, United States*

‖*Current address: Department of Veterinary Integrative Biosciences, School of Veterinary Medicine and Biomedical Sciences, Texas A&M University, College Station, Texas 77843, United States*

E-mail: pbs13@pitt.edu

Table S1: T1 diagnostic values for all systems studied using CCSD(T). T1 value ≤ 0.05 indicates single-reference character for a transition metal complex.[1]

Structure	T1 diagnostic
1	0.02
2	0.03
2*	0.04
TS(2*-3*)	0.06
3	0.02
3*	0.02
TS(3*-4*)	0.03
4*	0.03
4	0.02
5	0.04
5.1	0.02
TS(5.1-5.2)	0.04
5.2	0.05
TS(5.2-5.3)	0.07
5.3	0.06
TS(5.3-5.4)	0.04
5.4	0.03
TS(5.4-5.5)	0.04
5.5	0.03
TS(5.5-5.6)	0.04
5.6	0.02
6	0.03
6*	0.02
6.1	0.02
TS(6.1-7)	0.03
7	0.03
8	0.03
TS(8-8.1)	0.03
8.1	0.02
TS(8.1-8.2)	0.03
8.2	0.03
8.3	0.03
TS(8.3-9)	0.03
9	0.02
TS(9-9.1)	0.02
9.1	0.02
TS(9.1-9.2)	0.03
9.2	0.02
TS(9.2-9.3)	0.03
9.3	0.02

Table S2: Adsorption energies for reactions 1 → 2, reactions 4 → 5, and reactions 7 → 8, forward (Vf) and reverse (Vr) barriers (in kJ/mol) for reactions 2 → 3, 3 → 4, 5.4 → 5.6, 6* → 7, 8 → 9, and 9 → 9.3 computed with LDA, LDA@FLOSIC, FLOSIC, LSIC(z_σ), PBE, r^{2}SCAN, and CCSD(T) energies. Reaction 6* → 6.1 is an adsorption step. The LDA@FLOSIC, FLOSIC, and LSIC(z_σ) values correspond to the lowest-energy FLOSIC-LDA solutions identified.

		LDA	LDA@FLOSIC	FLOSIC	LSIC(z_σ)	PBE	r^{2}SCAN	CCSD(T)
step (i)	1 → 2	−168.24	−53.40	−376.57	−82.26	−122.63	−144.54	−65.16
step (ii)	Vf(2-3)	74.17	83.76	179.50	154.09	78.33	81.69	95.84
	Vr(2-3)	328.48	479.12	550.94	467.97	289.40	314.90	373.18
step (iii)	Vf(3-4)	71.23	119.48	121.82	199.15	111.94	119.59	160.39
	Vr(3-4)	76.71	38.11	31.42	108.51	116.09	105.94	132.20
step (iv)	4 → 5	−124.27	−63.56	−72.79	13.76	−77.19	−69.98	−37.42
step (v)	Vf(5.4-5.5)	135.03	148.58	165.30	162.07	145.34	155.55	150.27
	Vr(5.4-5.5)	97.21	108.96	195.40	174.58	114.11	130.76	143.29
	Vf(5.5-5.6)	51.57	142.75	220.97	106.90	22.19	44.62	83.46
	Vr(5.5-5.6)	263.32	390.83	433.70	436.96	283.79	325.89	422.82
step (vi)	6 + NO(g) → 6*	−121.51	71.66	68.36	143.51	−75.62	−60.14	−5.54
step (vi)	6* → 6.1	−50.61	−116.28	−88.17	−74.28	−38.39	−46.77	−38.90
	Vf(6.1-7)	27.19	42.75	44.17	51.50	32.12	36.67	35.16
	Vr(6.1-7)	45.63	27.61	53.26	30.51	37.49	41.64	45.98
step (vii)	7 → 8	−106.39	−112.35	−116.91	−33.68	−65.87	−74.74	−77.50
step (viii)	Vf(8-8.1)	38.64	18.82	31.37	20.62	30.73	28.57	36.60
	Vr(8-8.1)	23.62	28.44	9.25	25.41	25.41	29.70	27.26
	Vf(8.1-8.2)	95.82	63.47	105.91	87.11	104.33	112.21	106.75
	Vr(8.1-8.2)	47.35	48.44	43.14	70.90	60.00	56.38	63.43
	8.2-8.3	42.33	91.36	−31.51	−4.28	16.95	17.09	21.59
	Vf(8.3-9)	6.78	−2.87	35.14	9.79	12.20	14.78	3.79
	Vr(8.3-9)	54.58	19.36	−22.52	−1.61	50.59	45.28	51.86
step(ix)	Vf(9-9.1)	110.99	107.17	162.61	148.74	126.90	137.26	142.97
	Vr(9-9.1)	90.83	88.97	156.23	144.68	107.81	125.32	140.99
	Vf(9.1-9.2)	127.15	104.48	154.48	124.67	125.75	141.98	179.30
	Vr(9.1-9.2)	118.24	91.09	126.89	110.12	121.01	132.61	173.09
	Vf(9.2-9.3)	69.39	49.61	66.03	52.16	61.26	57.96	79.26
	Vr(9.2-9.3)	347.83	309.86	271.35	312.59	346.69	366.13	431.02

Table S3: Individual errors in forward (Vf) and reverse (Vr) reaction barriers relative to CCSD(T) energies, along with mean absolute error (MAE), and mean error (ME) (in kJ/mol) for LDA, LDA@FLOSIC, FLOSIC, LSIC(z_σ), PBE, and r^{2}SCAN for reactions 2 → 3, 3 → 4, 5.4 → 5.6, 6.1 → 7, 8 → 9, and 9 → 9.3. Reactions 6 + NO(g) → 6* and 6* → 6.1 are not included as they are adsorption steps. Errors for LDA@FLOSIC, FLOSIC, and LSIC(z_σ) errors correspond to the lowest-energy FLOSIC-LDA solutions identified.

		LDA	LDA@FLOSIC	FLOSIC	LSIC(z_σ)	PBE	r^{2}SCAN
step (i)	1 → 2	−103.08	11.76	−311.51	−17.10	−57.47	−79.38
step (ii)	Vf(2-3)	−21.67	−12.09	83.66	58.25	−17.51	−14.15
	Vr(2-3)	−44.70	105.94	177.76	94.79	−83.78	−58.28
step (iii)	Vf(3-4)	−89.16	−40.91	−38.57	38.76	−48.45	−40.80
	Vr(3-4)	−55.49	−94.09	−100.78	−23.69	−16.12	−26.26
step (iv)	4 → 5	−86.85	−26.15	−35.38	51.18	−39.77	−32.56
step (v)	Vf(5.4-5.5)	−15.24	−1.69	15.03	11.80	−4.94	5.28
	Vr(5.4-5.5)	−46.08	−34.33	52.11	31.30	−29.18	−12.53
	Vf(5.5-5.6)	−31.89	59.28	137.51	23.44	−61.28	−38.84
	Vr(5.5-5.6)	−159.50	−31.99	10.88	14.14	−139.03	−96.93
step (vi)	6 + NO(g) → 6*	−115.98	77.20	73.89	149.05	−70.08	−54.61
	6* → 6.1	−11.71	−77.39	−49.27	−35.39	0.50	−7.87
	Vf(6.1-7)	−7.97	7.59	9.01	16.34	−3.04	1.51
	Vr(6.1-7)	−0.34	−18.36	7.28	−15.47	−8.49	−4.34
step (vii)	7 → 8	−28.89	−34.85	−39.41	43.82	11.63	2.76
step (viii)	Vf(8-8.1)	2.03	−17.78	−5.23	−15.99	−5.87	−8.04
	Vr(8-8.1)	−3.64	1.18	−18.01	−1.85	−1.85	2.44
	Vf(8.1-8.2)	−10.93	−43.28	−0.84	−19.63	−2.42	5.47
	Vr(8.1-8.2)	−16.07	−14.98	−20.29	7.48	−3.43	−7.04
	Vf(8.3-9)	2.99	−6.66	31.34	6.00	8.40	10.99
	Vr(8.3-9)	2.73	−32.50	−74.38	−53.46	−1.26	−6.58
step (ix)	Vf(9-9.1)	−31.98	−35.79	19.64	5.77	−16.06	−5.71
	Vr(9-9.1)	−50.16	−52.02	15.23	3.69	−33.18	−15.67
	Vf(9.1-9.2)	−52.15	−74.82	−24.82	−54.63	−53.55	−37.32
	Vr(9.1-9.2)	−54.85	−82.00	−46.20	−62.97	−52.08	−40.49
	Vf(9.2-9.3)	−9.87	−29.66	−13.23	−27.11	−18.00	−21.31
	Vr(9.2-9.3)	−83.19	−121.16	−159.67	−118.43	−84.33	−64.89
	MAE (steps (ii), (iii), (v))	57.97	47.54	77.04	37.02	50.04	36.63
	ME (steps (ii), (iii), (v))	−57.97	−6.23	42.20	31.10	−50.04	−35.31
	MAE (steps (vi), (viii), (ix))	23.49	38.41	31.80	29.20	20.85	16.56
	ME (steps (vi), (viii), (ix))	−22.39	−37.16	−20.01	−23.59	−19.65	−13.64
	MAE (overall reaction barriers)	36.03	41.73	48.25	32.04	31.47	23.86
	ME (overall reaction barriers)	−35.32	−25.91	2.61	−3.70	−30.70	−21.52

Table S4: Energy differences (in kJ/mol) between two local FLOSIC-LDA minima—specifically, between the lower-energy and higher-energy FLOSIC-LDA solutions—computed using LDA@FLOSIC, FLOSIC, and LSIC(z_σ) energies for structures 6*, 8, 9.1, 9.2, TS(9.2-9.3), and 9.3. Structure 6* is a vdW complex of structure 6 and NO gas.

	LDA@FLOSIC	FLOSIC	LSIC(z_σ)
6*	145.40	−258.41	−4.10
8	133.35	−198.76	−9.04
9	170.45	−160.81	59.87
9.1	167.03	−146.00	62.65
9.2	178.05	−127.93	83.62
TS(9.2-9.3)	135.94	−190.70	32.36
9.3	216.20	−76.18	119.89

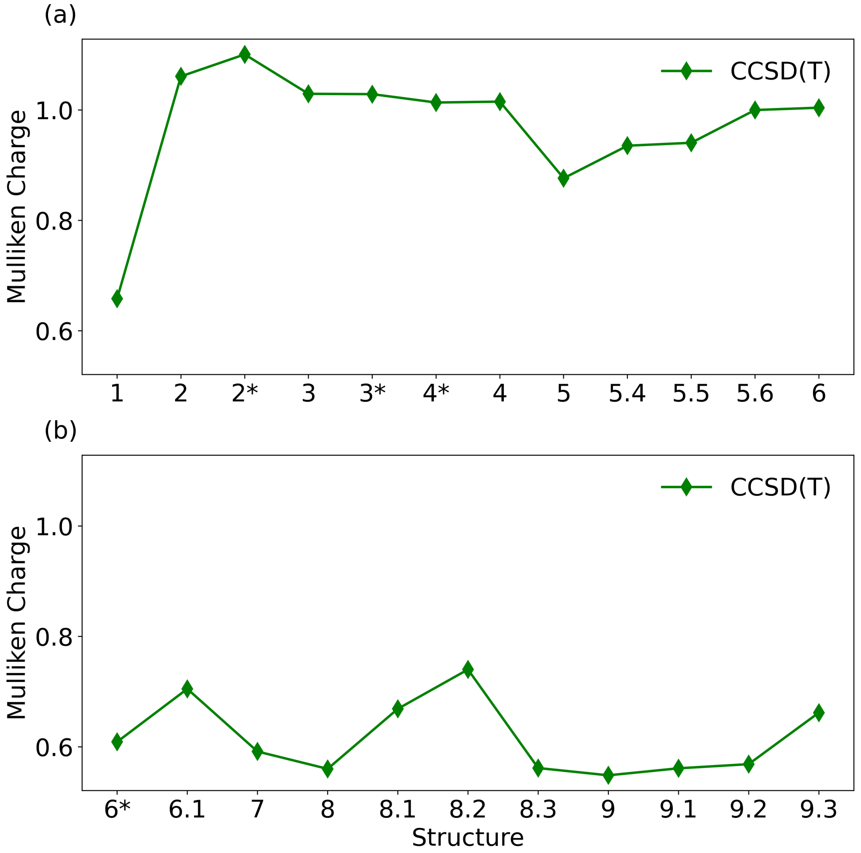

Figure S1: CCSD(T) Cu Mulliken side in (a) structure 1 and the oxidation side (structures 2 to 6), and (b) the reduction side (structures 6* to 9.3).

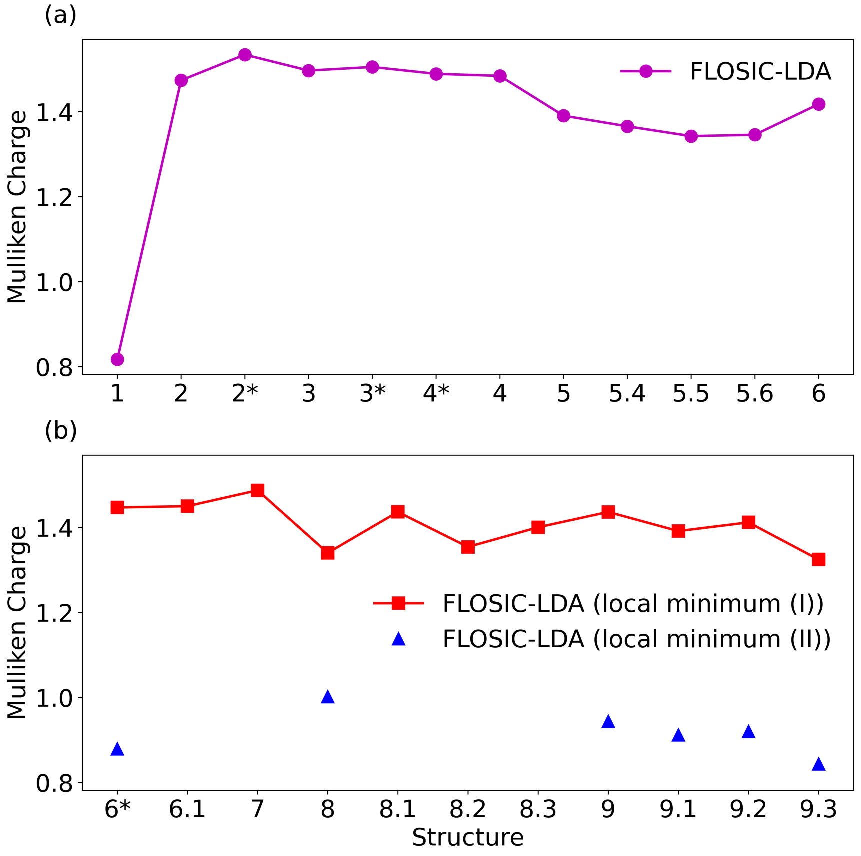

Figure S2: FLOSIC-LDA Cu Mulliken charge in (a) structure 1 and the oxidation side (structures 2 to 6), shown in (magenta, circles), and (b) the reduction side (structures 6* to 9.3). There are two FOD local minima obtained for structures 6*, 8, 9, 9.1, 9.2, and 9.3. The Cu charges for these two minima are shown in (red,squares) and (blue, triangles).

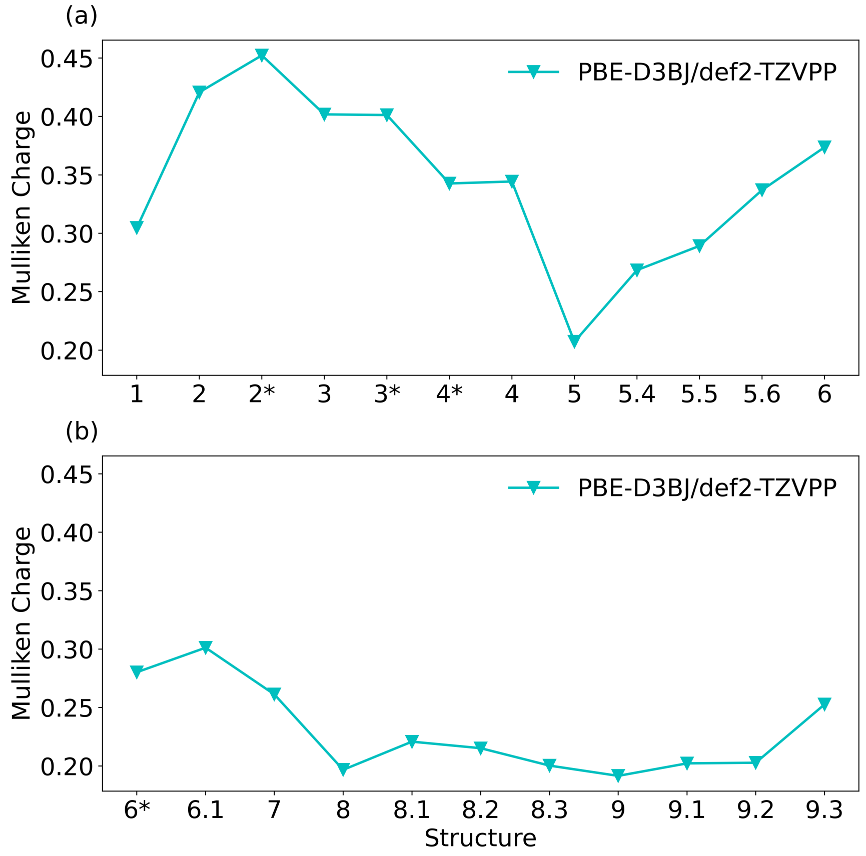

Figure S3: PBE-D3BJ/def2-TZVPP Cu Mulliken charge in (a) structure 1 and the oxidation side (structures 2 to 6), and (b) the reduction side (structures 6* to 9.3).

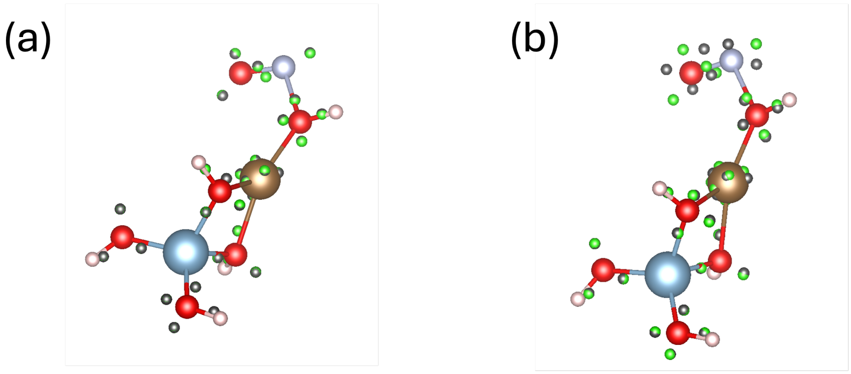

Figure S4: Structure 6*: Two local FLOSIC-LDA minima corresponding to (a) higher-energy and (b) lower-energy FOD configurations. Structure 6* is a vdW complex of structure 6 and NO gas. Color scheme: Cu (brown), Al (silver), O (red), H (pink), up-spin FOD (green), down-spin FOD (black).

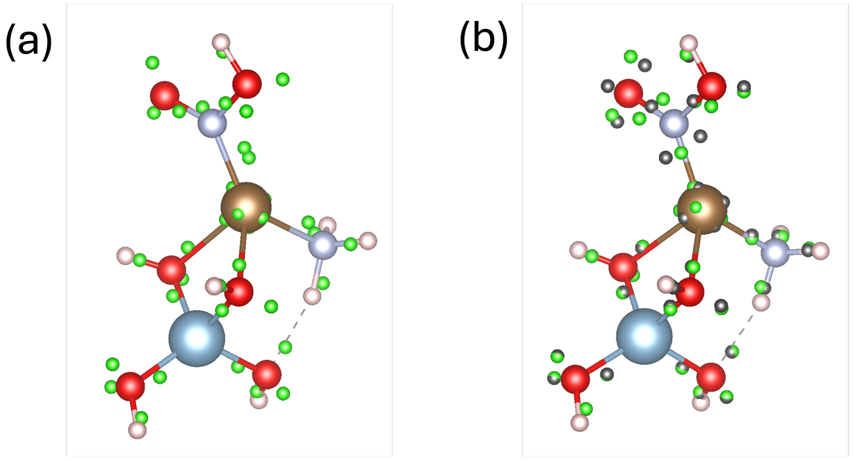

Figure S5: Structure 8: Two local FLOSIC-LDA minima corresponding to (a) higher-energy and (b) lower-energy FOD configurations. Color scheme: Cu (brown), Al (silver), O (red), H (pink), up-spin FOD (green), down-spin FOD (black).

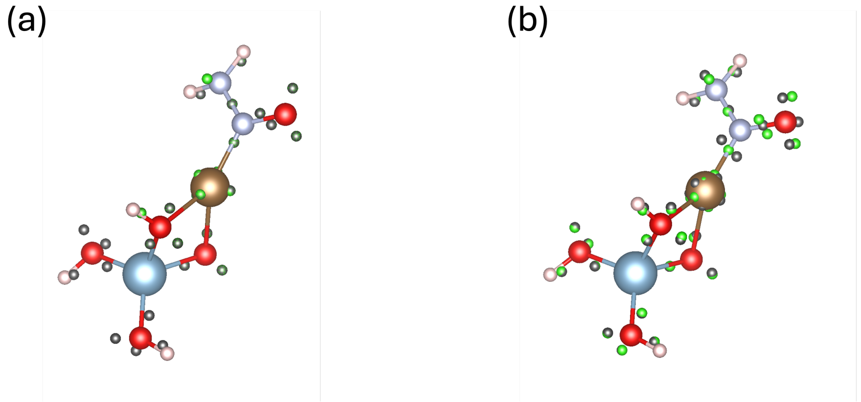

Figure S6: Structure 9: Two local FLOSIC-LDA minima corresponding to (a) higher-energy and (b) lower-energy FOD configurations. Color scheme: Cu (brown), Al (silver), O (red), H (pink), up-spin FOD (green), down-spin FOD (black).

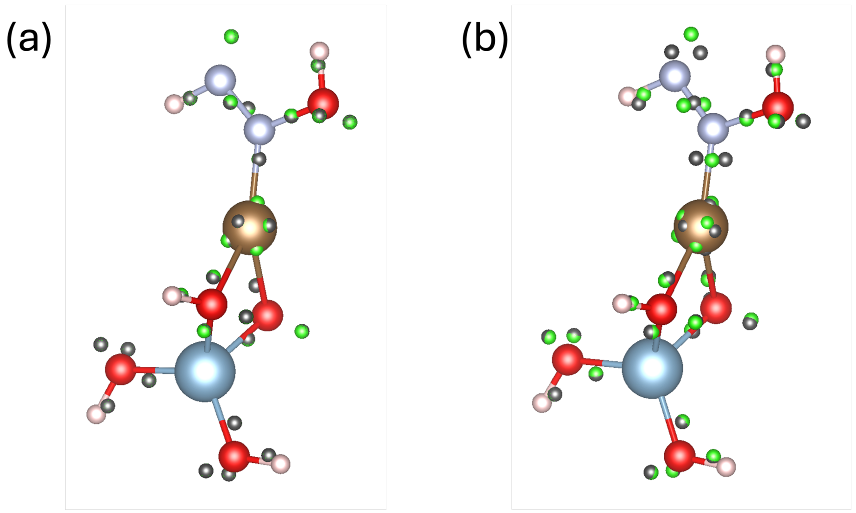

Figure S7: Structure 9.1: Two local FLOSIC-LDA minima corresponding to (a) higher-energy and (b) lower-energy FOD configurations. Color scheme: Cu (brown), Al (silver), O (red), H (pink), up-spin FOD (green), down-spin FOD (black).

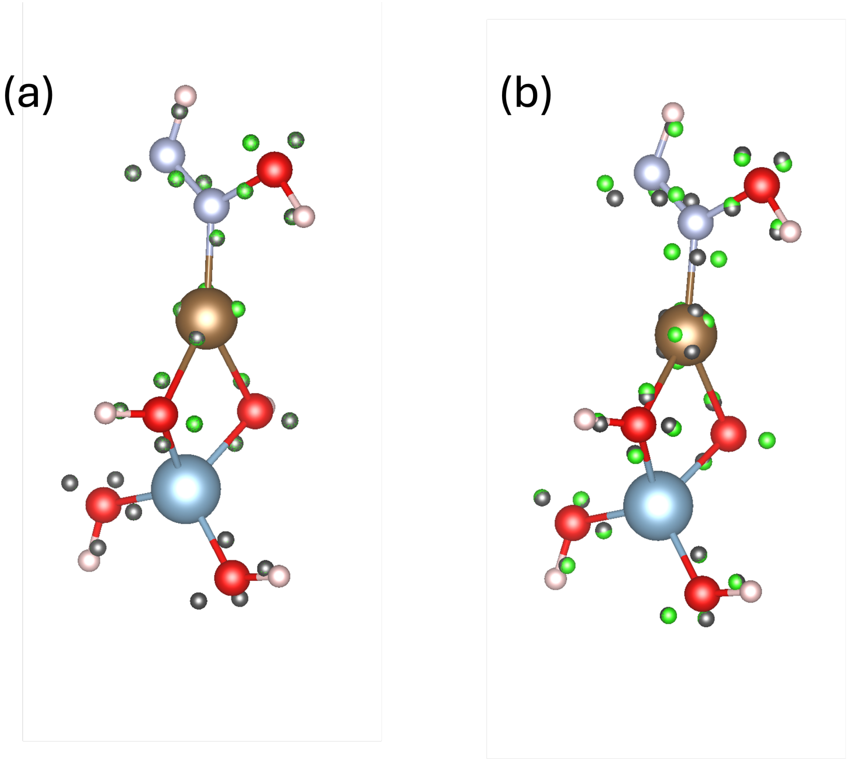

Figure S8: Structure 9.2: Two local FLOSIC-LDA minima corresponding to (a) higher-energy and (b) lower-energy FOD configurations. Color scheme: Cu (brown), Al (silver), O (red), H (pink), up-spin FOD (green), down-spin FOD (black).

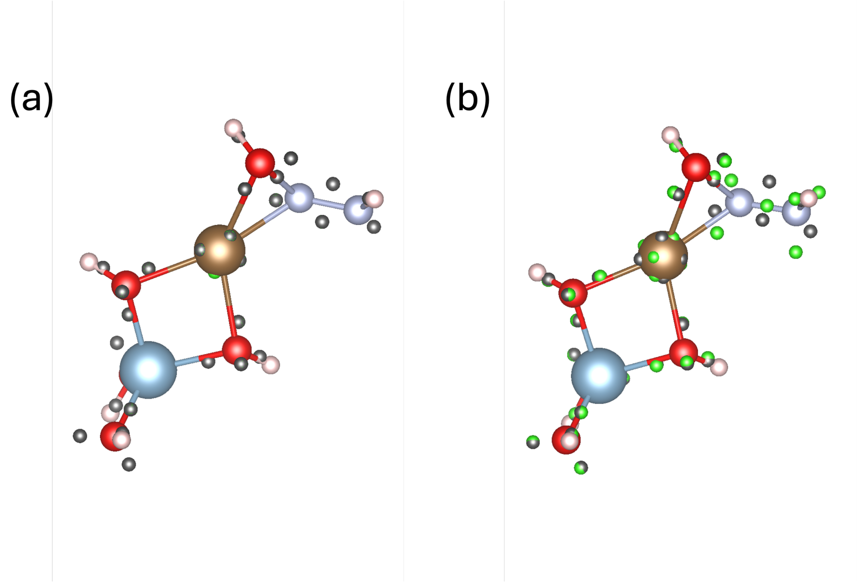

Figure S9: Structure TS(9.2-9.3): Two local FLOSIC-LDA minima corresponding to (a) higher-energy and (b) lower-energy FOD configurations. Color scheme: Cu (brown), Al (silver), O (red), H (pink), up-spin FOD (green), down-spin FOD (black).

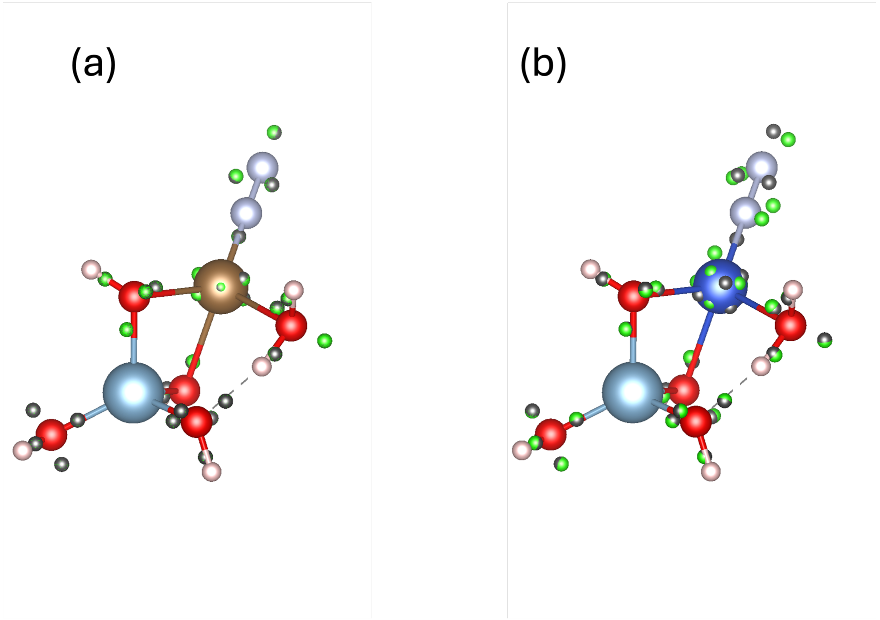

Figure S10: Structure 9.3: Two local FLOSIC-LDA minima corresponding to (a) higher-energy and (b) lower-energy FOD configurations. Color scheme: Cu (brown), Al (silver), O (red), H (pink), up-spin FOD (green), down-spin FOD (black).

Table S5: Individual errors, mean absolute errors (MAE), and mean error (ME) in LDA, LDA@FLOSIC, FLOSIC-LDA, and LSIC(z_σ) adsorption energies ($6^* \rightarrow 6.1$, $7 \rightarrow 8$) and forward (Vf) and reverse (Vr) barriers using the metastable ($3d^{10}$) and lowest-energy ($3d^9$) FLOSIC-LDA FOD solutions for structures 6*, 8, 9, 9.1, 9.2, TS(9.2-9.3), and 9.3. The rest of the reacting species are in lowest-energy ($3d^9$) FOD solutions in the table. All errors are are reported relative to CCSD(T) energies (in kJ/mol). Structure 6* is a vdW complex of structure 6 and NO gas.

		LDA	LDA@FLOSIC	FLOSIC	LSIC(z_σ)
$3d^{10} \rightarrow 3d^9$	$6^* \rightarrow 6.1$	−11.71	68.01	−307.68	−39.48
$3d^9 \rightarrow 3d^9$		−11.71	−77.39	−49.27	−35.39
$3d^9 \rightarrow 3d^{10}$	$7 \rightarrow 8$	−28.89	−168.21	159.35	52.86
$3d^9 \rightarrow 3d^9$		−28.89	−34.85	−39.41	43.82
$3d^{10} \rightarrow 3d^9$	Vf(8-8.1)	2.03	115.57	−203.99	−25.03
$3d^9 \rightarrow 3d^9$		2.03	−17.78	−5.23	−15.99
$3d^9 \rightarrow 3d^{10}$	Vr(8.3-9)	2.73	137.95	−235.18	6.41
$3d^9 \rightarrow 3d^9$		2.73	−32.50	−74.38	−53.46
$3d^{10} \rightarrow 3d^{10}$ (TS $3d^9$)	Vf(9-9.1)	−31.98	134.65	−141.16	65.64
	Vr(9-9.1)	−50.16	115.00	−130.76	66.34
$3d^9 \rightarrow 3d^9$	Vf(9-9.1)	−31.98	−35.79	19.64	5.77
	Vr(9-9.1)	−50.16	−52.02	15.23	3.69
$3d^{10} \rightarrow 3d^{10}$ (TS $3d^9$)	Vf(9.1-9.2)	−52.15	92.20	−170.82	8.02
	Vr(9.1-9.2)	−54.85	96.05	−174.14	20.65
$3d^9 \rightarrow 3d^9$	Vf(9.1-9.2)	−52.15	−74.82	−24.82	−54.63
	Vr(9.1-9.2)	−54.85	−82.00	−46.20	−62.97
$3d^{10} \rightarrow 3d^{10}$	Vf(9.2-9.3)	−9.87	12.46	49.54	24.15
	Vr(9.2-9.3)	−83.19	−40.89	−45.15	−30.89
$3d^9 \rightarrow 3d^9$	Vf(9.2-9.3)	−9.87	−29.66	−13.23	−27.11
	Vr(9.2-9.3)	−83.19	−121.16	−159.67	−118.43
	MAE (metastable)	32.76	98.10	161.78	33.95
	ME	−31.80	56.28	−120.00	14.87
	MAE (lowest-energy)	32.76	55.80	44.71	42.13
	ME	−31.80	−55.80	−37.73	−31.47

References

(1) Jiang, W.; DeYonker, N. J.; Wilson, A. K. Multireference character for 3d transition-metal-containing molecules. *Journal of chemical theory and computation* **2012**, *8*, 460–468.
